# Exclusion of dysfunctional mitochondria from Balbiani body during early oogenesis of *Thermobia*

**DOI:** 10.1007/s00441-016-2414-x

**Published:** 2016-05-10

**Authors:** Waclaw Tworzydlo, Elzbieta Kisiel, Wladyslawa Jankowska, Alicja Witwicka, Szczepan M. Bilinski

**Affiliations:** Department of Developmental Biology and Morphology of Invertebrates, Institute of Zoology, Jagiellonian University, Krakow, Poland

**Keywords:** Oogenesis, Balbiani body, Mitochondrial dynamics, Mitochondrial network

## Abstract

Oocytes of many invertebrate and vertebrate species contain a characteristic organelle complex known as the Balbiani body (Bb). Until now, three principal functions have been ascribed to this complex: delivery of germ cell determinants and localized RNAs to the vegetal cortex/posterior pole of the oocyte, transport of the mitochondria towards the germ plasm, and participation in the formation of lipid droplets. Here, we present the results of a computer-aided 3D reconstruction of the Bb in the growing oocytes of an insect, *Thermobia domestica.* Our analyses have shown that, in *Thermobia*, the central part of each fully developed Bb comprises a single intricate mitochondrial network. This “core” network is surrounded by several isolated bean-shaped mitochondrial units that display lowered membrane potential and clear signs of degeneration. In light of the above results and recent theoretical models of mitochondrial quality control, the role of the Bb is discussed. We suggest that, in addition to the aforementioned functions, the Bb is implicated in the selective elimination of dysfunctional mitochondria during oogenesis.

**Electronic supplementary material:**

The online version of this article (doi:10.1007/s00441-016-2414-x) contains supplementary material, which is available to authorized users.

## Introduction

Early oocytes of many invertebrate and vertebrate species contain a transient assemblage (complex) of organelles known as the Balbiani body (Bb) or mitochondrial cloud. The structure and composition of the Bb differ even between related species and are highly dynamic during subsequent stages of Bb morphogenesis, i.e. its formation, gradual development and ultimate fragmentation (Heasman et al. 1984; Cox and Spradling 2003; Marlow and Mullins 2008; reviewed in Kloc et al. 2004a). Despite this variability, Bbs always contain two essential elements: numerous tightly clustered mitochondria, and electron-dense granulo-fibrillar material, termed the nuage (composition and functioning of nuage is discussed in Jaglarz et al. 2011; Kloc et al. 2014). The Bbs may also comprise centrioles, Golgi complexes (dictyosomes), and elements of rough (coated with ribosomes) or smooth (lacking bound ribosomes) elements of endoplasmic reticulum (Kloc et al. 2004a, 2014). Surprisingly, the 3-dimensional (3D) organization of the Bb has never been analyzed, and as a result exact relationships between its constituents remain unknown. In spite of relatively numerous studies, the functioning of the Bb during oocyte growth is still incompletely understood. Until now, three principal functions have been ascribed to this organelle complex: delivery of germ cell determinants and localized mRNAs to the oocyte vegetal/posterior pole (Kloc and Etkin 1995; Kloc et al. 1996, 2001; Wilk et al. 2004), transport of the mitochondria to the germ plasm or vegetal cortex (Tourte et al. 1984; Kloc and Etkin 1995; Kloc et al. 1996, 2001), and a participation in the formation of lipid droplets (Jedrzejowska and Kubrakiewicz 2010). It has been suggested, in this context, that the Bb represents an evolutionary ancestral organelle involved in the localization and/or enrichment of maternal macromolecules and organelles in certain oocyte regions (reviewed by Kloc et al. 2014).

Recent studies of the morphology and behavior of mitochondria in yeast and cultured cells have delivered important findings (reviewed by Mitra 2013; van der Bliek et al. 2013) that are essential for understanding the functioning of these organelles during the formation and fragmentation of the Bb. Firstly, mitochondria are highly dynamic organelles that constantly fuse and divide. The actual morphology of mitochondria in differentiating or proliferating cells depends on a balance of the two opposed processes: mitochondrial fusion and fission (collectively termed mitochondrial dynamics). Disruption of mitochondrial fusion maintains mitochondria in a fragmented state (as solitary bean- or rod-shaped organelles), whereas obliterating fission events leads to the formation of extensive mitochondrial networks. Secondly, the mitochondrial dynamics apparently contributes to the maintenance and inheritance of mitochondrial DNA (mtDNA) through two activities: rescue of damaged mitochondria by fusion, and elimination of non-functional organelles by fission (Parone et al. 2008; Westermann 2010; Youle and van der Bliek 2012; Ni et al. 2015). The latter process is linked with mitophagy, i.e. autophagic elimination of old and dysfunctional mitochondria or “mitochondrial parts”. Thirdly, it has been shown that mtDNA with severe mutations can be selectively eliminated during oogenesis (Fan et al. 2008; Stewart et al. 2008). The mechanisms of this purifying selection are not yet clear; it has been hypothesized, however, that they include such processes as: elimination of dysfunctional mitochondria by mitophagy, expansion of healthy mitochondria within the ooplasm, and apoptosis of oocytes with excessive deleterious mtDNA mutations (Fan et al. 2008; Stewart et al. 2008).

To gain an insight into the function of the Bb, we decided to reconstruct its 3D organization at the level of electron microscopy (EM). In our analyses, developing oocytes of the basally branching “apterygotous” insect, *Thermobia domestica*, were exploited. There were three reasons for such a selection: (1) the Bb of *Thermobia* is relatively small and consists of a limited number of organelles that obviously facilitate 3D analyses, especially on the EM level; (2) early stages of oogenesis of this species are greatly simplified and do not involve formation of germline cysts; (3) the oocytes and eggs of *Thermobia* are roughly spherical and do not contain any specialized region of the cytoplasm, e.g. germ plasm or oosome, implying that the Bb in this species cannot be involved in the localization of maternal organelles/macromolecules to certain oocyte regions.

Insect ovaries are composed of several functional units termed ovarioles. As a rule, the ovariole consists of three elements, the terminal filament, germarium and vitellarium. The terminal filament is a simple stack of disc-shaped somatic cells, the germarium contains dividing and differentiating oogonia, whereas the vitellarium comprises several developing ovarian follicles in a linear arrangement (see Buning 1994; Bilinski 1998, for further details). Traditionally, two fundamental types of insect ovaries are distinguished: meroistic and panoistic. In meroistic ovaries, the oocytes are associated with highly active and polyploid supporting cells, termed the nurse cells, while in panoistic ovaries these cells are absent (Buning 1994; Bilinski 1998). The ovaries of *Thermobia* are panoistic, and differ substantially from those of *Drosophila melanogaster*. In the fruit fly, asymmetric divisions of germline stem cells, located at the tip of the germarium, generate progenitor cells, termed the cystoblasts. Then, each cystoblast divides mitotically four times to form a single germline cyst composed of 16 sibling cells (cystocytes). Only 1 of these cells differentiates into the oocyte, the remaining 15 transform into supporting nurse cells (Fuller and Spradling 2007). In *Thermobia*, the cystoblasts never divide mitotically, instead they directly enter the meiotic prophase and become the oocytes. It was hypothesized in this context that in *Thermobia* (and presumably also in other basally branching insects with panoistic ovaries), the syncytial phase of oogenesis has been lost (eliminated) during evolution (Tworzydlo et al. 2014). Our previous analyses have shown additionally that in each oocyte, during the so-called bouquet stage of the meiotic prophase, the Bb is present. Interestingly, the Bb is invariably located next to this segment of the nuclear envelope to which the telomeres of the bouquet chromosomes are attached. This observation led to the idea that the localization of the Bb together with polar attachment of the bouquet chromosomes play a crucial role in the asymmetrization of *Thermobia* oocytes (Tworzydlo et al. 2014).

Here, we show that in *Thermobia* ovaries, the Bb is formed as early as in the cystoblast, attains maximal dimensions during the meiotic prophase, and disperses at the onset of previtellogenic oocyte growth. Surprisingly, during the entire prophase of meiosis, mitochondria of the Bb form a hyperfused network. We also show that this mitochondrial network is always surrounded by small isolated mitochondria, and that some of them apparently degenerate. We postulate in this context that the Bb of *Thermobia* is involved in a selective elimination of defective mitochondria and can contribute to the inheritance of mtDNA. Finally, our analyses indicate that some early germline cells (the cystoblasts and meiotic oocytes) are eliminated via apoptosis, and that the Bbs in apoptotic cells exhibit altered morphology. This result, in turn, suggests that two mechanisms play a role in purifying selection of mtDNA: elimination of defective mitochondria in the oocytes (with involvement of the Bb) and elimination of early germline cells overloaded with dysfunctional mitochondria.

## Materials and methods

### Animals

Cultures of the firebrat, *Thermobia domestica* (Packard, 1873) were maintained at 37 °C and 60 % relative humidity (RH) in plastic boxes containing test tubes filled with water and mixed oat flakes, powder milk and dried water fleas (see Kisiel and Klag 2001 for further details).

### Light and electron microscopy

The ovaries were dissected under a Nikon SMZ1500 stereoscopic microscope (Nikon, Japan). They were fixed in a mixture of 2 % formaldehyde and 2.5 % glutaraldehyde in 0.1 M phosphate buffered saline (PBS), pH 7.3, for several days. Isolated ovarioles were rinsed and postfixed in 2 % osmium tetroxide and 0.8 % potassium ferrocyanide in the same buffer for 30 min at 4 °C. After dehydration in a series of ethanol and acetone, the material was embedded in Glycid Ether 100 (Epon 812) resin (Serva, Heidelberg, Germany). Semithin sections (0.7 μm thick) were stained with 1 % methylene blue and examined under a Leica DMR (Heidelberg, Germany) or Nikon Eclipse Ni (Nikon) light microscopes. Ultrathin sections (80 nm thick) were contrasted with uranyl acetate and lead citrate according to standard protocols and analyzed with a Jeol JEM 2100 transmission electron microscope (TEM) at 80 kV.

### DNA localization

Dissected ovaries were fixed in a mixture of 3 % formaldehyde and 1.5 % glutaraldehyde in PBS at room temperature for 1 h. After dehydration in a series of ethanol, the material was embedded in Histocryl (Agar Scientific, Stansted, Essex, UK). Semithin sections were stained with Hoechst 33342 (1 μg/ml; Molecular Probes, Eugene, OR, USA) in the dark for 40 min, and analyzed with a Leica DMR fluorescence microscope, equipped with appropriate filters.

### 3D reconstruction of the Balbiani body

To reconstruct *Thermobia* 3D organization of the Bb in various developmental stages, we used serial sections of 5 early meiotic and 5 previtellogenic oocytes dissected from two young females. The EM microphotographs of these sections were scanned, *Thermobia* chosen organelles were contoured using CorelDRAW^®^ and *Thermobia* obtained images were processed with ImageJ software (Schneider et al. 2012) equipped with 3D viewer and Z-projection plugins.

### Analysis of mitochondrial activity

The ovaries were dissected in Grace’s Insect Medium (Sigma, St. Louis, MO, USA) and incubated with MitoTracker Deep Red^®^ (Molecular Probes) for 30 min at 37 °C in the dark. MitoTracker stock solution was prepared by dissolving 50 μg MitoTracker in 50 ml DMSO. To get the final concentration, the stock solution was diluted 1:200 with Grace’s Insect Medium. After incubation, the material was rinsed twice in Grace’s Insect Medium and fixed in 4 % formaldehyde for 30 min. Then, the ovaries were rinsed in PBS and stained with Hoechst 33342 for 30 min in the dark. After rinsing with PBS, the ovarioles were whole-mounted on microscope slides and examined with a Zeiss LSM 510 Meta confocal microscope.

### Detection of apoptotic cells

For detection of the apoptotic cells, we adapted the modified version of ApopTag_®_ Plus Peroxidase in Situ Apoptosis Detection Kit (Millipore, Billerica, MA, USA) which utilizes TUNEL assay. Briefly, the dissected ovarioles were fixed in 4 % formaldehyde for 1 h at room temperature and then washed twice, 15 min each, in PBS–0.1 % Triton X-100. They were incubated in proteinase K in PBS (50 μg/ml), washed twice in distilled water and incubated in 3 % H_2_O_2_ for 15 min. The material was then rinsed in PBS-0.1 % Triton X-100 and transferred into an equilibration buffer for 10 min. Subsequently, the ovarioles were incubated in terminal deoxynucleotidyl transferase (TdT) buffer for 1 h at 37 °C and transferred into stop/washing buffer for 10 min. After washing in PBS-0.1 % Triton X-100, they were incubated in anti-digoxigenin conjugate for 30 min, washed in PBS and labeled in DAB solution for 3 min. They were washed three times in distilled water, mounted on the microscopic slides and analyzed under a Leica DMR light microscope.

## Results

### Morphology of the ovariole

**Fig. 1:**
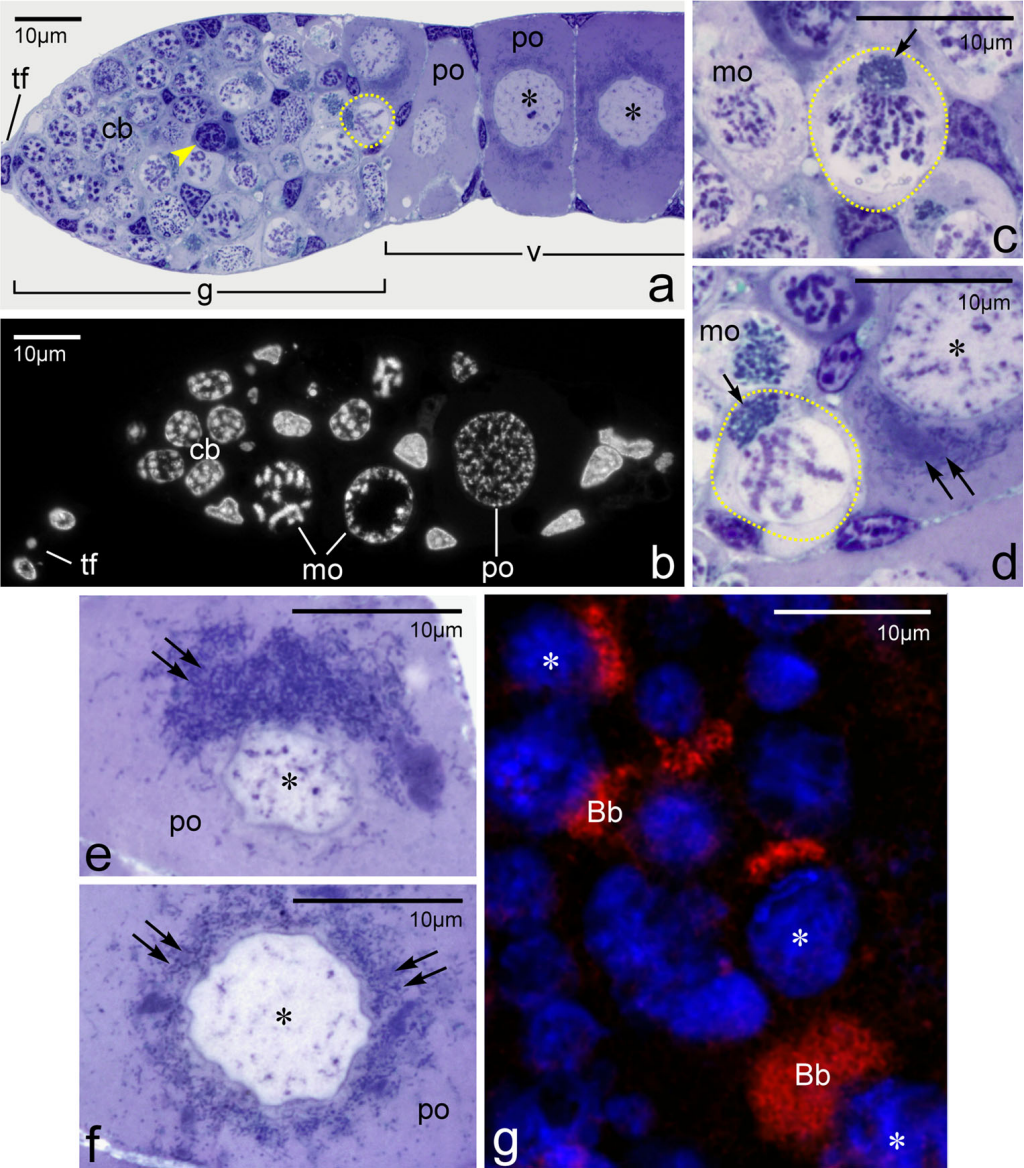
Ovariole and morphology of germline cells. **a**, **b** Longitudinal sections through the anterior part of an ovariole. **c**–**f** Higher magnifications of bouquet stage and previtellogenic oocytes.** a**,** c**–**f** Semithin sections stained with methylene blue;** b** histocryl section stained with Hoechst 33342. **g** Fragment of the germarium incubated with MitoTracker and counterstained with Hoechst 33342. Note highly positive Bbs (mitochondrial networks). Individual mitochondria located outside of Bbs display weaker fluorescence. Cystoblast (*cb*), germarium (*g*), meiotic oocytes (*mo*); oocyte nuclei (*asterisks*), previtellogenic oocytes (*po*), vitellarium (*v*), terminal filament (*tf*), apoptotic germ cell (*arrowhead*), oocytes in bouquet stage are encircled. Note that Bbs are compact in meiotic oocytes (*arrows*) and dispersed in previtellogenic oocytes (*double arrows*)

Each of the two ovaries of *Thermobia domestica* is composed of 5 synchronously developing panoistic ovarioles (Tworzydlo et al. 2014). The ovarioles are built of 3 elements: an anterior terminal filament, germarium and vitellarium (Fig. 1a, b). The structure and ultrastructure of the terminal filament and the germarium have been described elsewhere (Kisiel and Klag 2001; Tworzydlo et al. 2014). Here, we concentrate solely on the ultrastructure of the cystoblasts, early meiotic oocytes and young previtellogenic oocytes.

### Morphogenesis and ultrastructure of the Bb

**Fig. 2:**
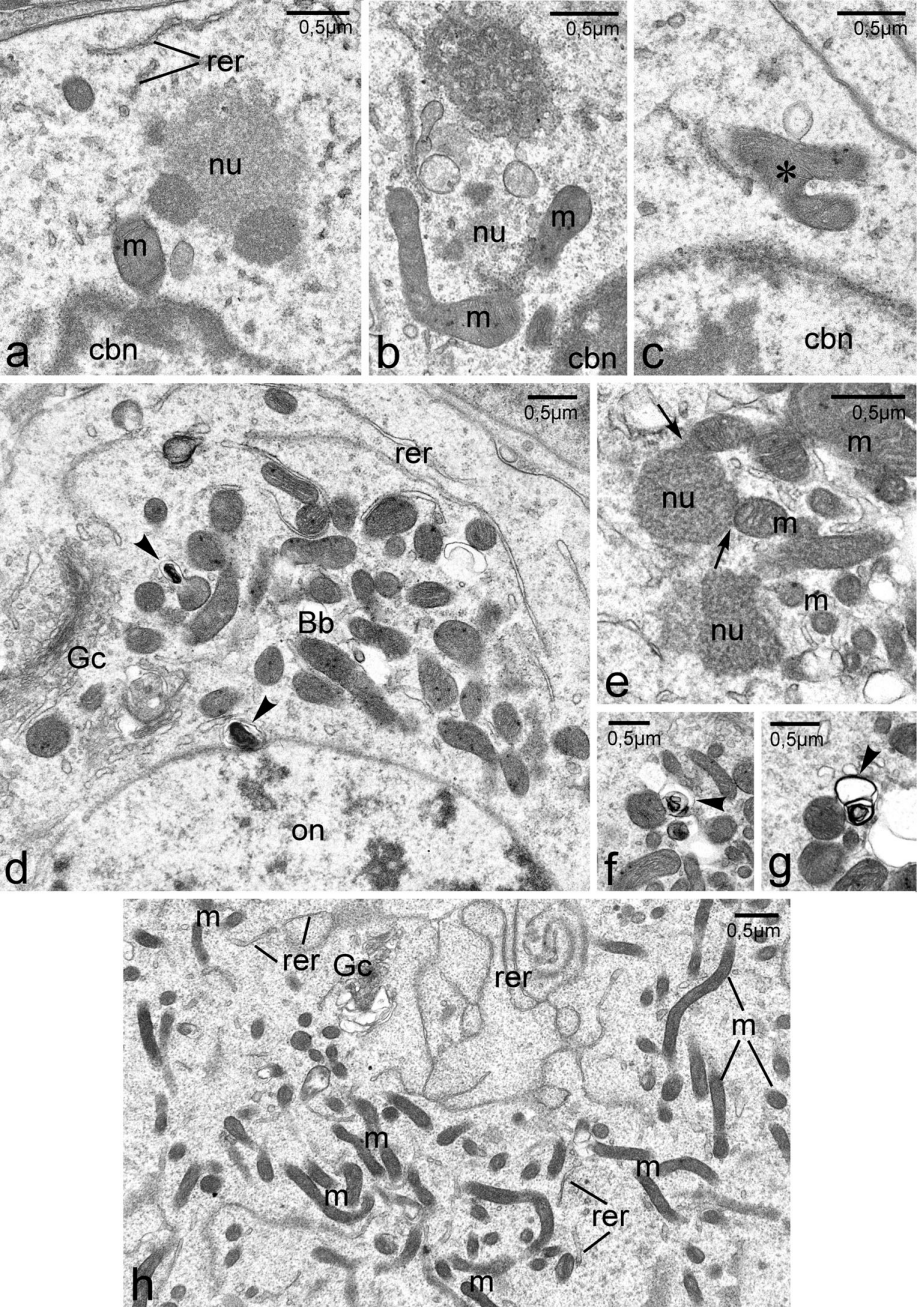
Ultrastructure and morphogenesis of the Balbiani body. **a**–**c** Cystoblasts (TEM). Accumulations of nuage (*nu*) are present in the neighborhood of nuclear envelope. Note variously shaped mitochondria located next to nuage accumulations. **d**, **e** Bb (*Bb*) in bouquet stage oocytes (TEM). **f**–**g** Degenerating mitochondria (TEM).** h** Perinuclear cytoplasm of a previtellogenic oocyte (TEM). Cystoblast nucleus (*cbn*), elements of rough endoplasmic reticulum (*rer*), Golgi complex (*Gc*), mitochondria (*m*), nuage accumulations (*nu*), oocyte nucleus (*on*). Note that some mitochondria are associated with nuage accumulations (**e**, *arrows*); degenerating mitochondria (**d**–**g**, *arrowheads*)

Our studies revealed that the Bbs start to form as early as in undifferentiated, premeiotic cystoblasts (Cbs). These cells reside in the anterior part of the germarium (Figs. 1a, b, 2a–c). They are equipped with large, roughly spherical nuclei containing prominent patches of heterochromatin in almost transparent karyoplasm. The cytoplasm of the Cbs comprises a small number of variously shaped mitochondria, single cisternae of the rough endoplasmic reticulum (RER), and conspicuous accumulations of the nuage (Fig. 2a, b). The Cbs’ mitochondria are morphologically not uniform: those that are in contact with nuage accumulations are elongated or even bifurcated (Fig. 2b, c, respectively), those located a distance from these accumulations are rod-shaped and often show signs of degradation (not shown). Although assemblages of the nuage and mitochondria in the Cbs are small and loosely arranged, we interpret them as initial Bbs.

The Bbs of early meiotic oocytes are much larger and more compact (Fig. 1c, d). Analysis of single ultrathin sections through the Bbs of bouquet stage oocytes revealed numerous profiles of mitochondria (see below), prominent accumulations of nuage, Golgi complexes and individual cisternae of the RER (Fig. 2d, e). The oocyte cytoplasm (ooplasm) contains, in addition to the Bb, free ribosomes, RER elements, and isolated bean-shaped mitochondria. Interestingly, these mitochondria show signs of degradation (see below). At the onset of previtellogenic growth, the Bb gradually disperses (disintegrates) surrounding the entire circumference of the oocyte nucleus (germinal vesicle) (Fig. 1e, f). Simultaneously, the oocyte mitochondria became highly elongated and filiform (Fig. 2h). Finally, during late previtellogenesis, mitochondria of the Bb populate the entire ooplasm (not shown).

### Are mitochondria of early meiotic oocytes physiologically equivalent?

To investigate whether mitochondria constituting the Bb network are physiologically different from individual ones that populate the ooplasm and surround the Bb, we incubated freshly dissected ovarioles in Grace’s Insect Medium with an addition of a fluorescent probe, MitoTracker. This experiment showed that the membrane potential of mitochondrial networks is high enough to be detected by this dye (Fig. 1g), whereas fluorescence displayed by individual mitochondria surrounding the Bbs is not as strong. This result suggests that individual mitochondria surrounding the Bb have distinctive physiological properties, and that presumably their membrane potential is lower.

### 3D organization of the Bb

**Fig. 3:**
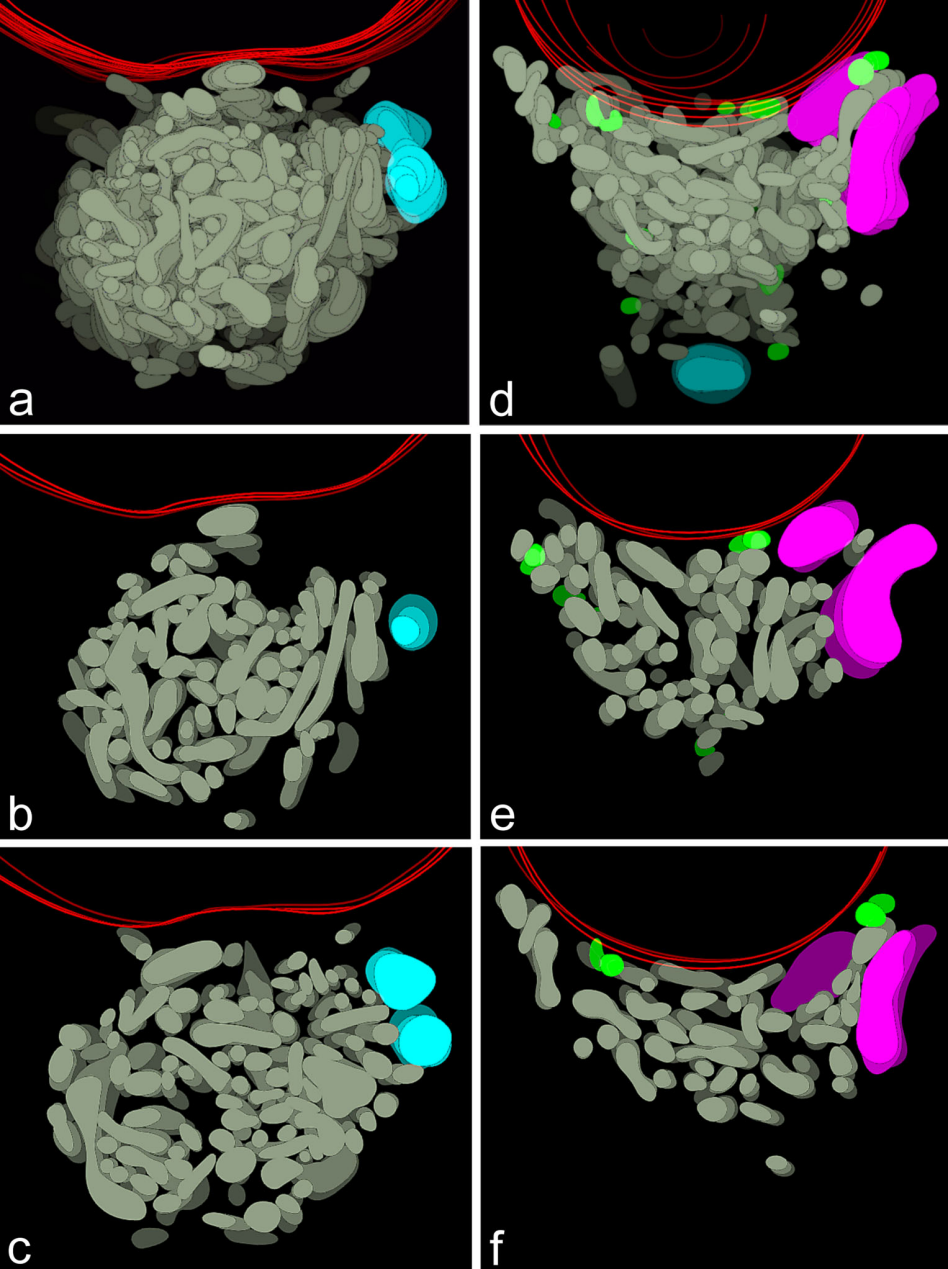
Computer-aided 3D reconstructions of Bbs. **a**, **d** Two representative 3D reconstructions of the Bb in bouquet stage oocytes. Mitochondrial network (*gray*), degenerating mitochondria (*green*), nuage accumulations (*blue*), Golgi complex (*magenta*), nuclear envelope (*red*). **b**,** c**, **e**,** f** Reconstructions showing central parts of (**a**) and (**d**), respectively. For these reconstructions, only 3 serial sections have been used. For clarity of the images, colors of subsequent (deeper and deeper) levels (representing subsequent sections) have been depicted darker

**Fig. 4:**
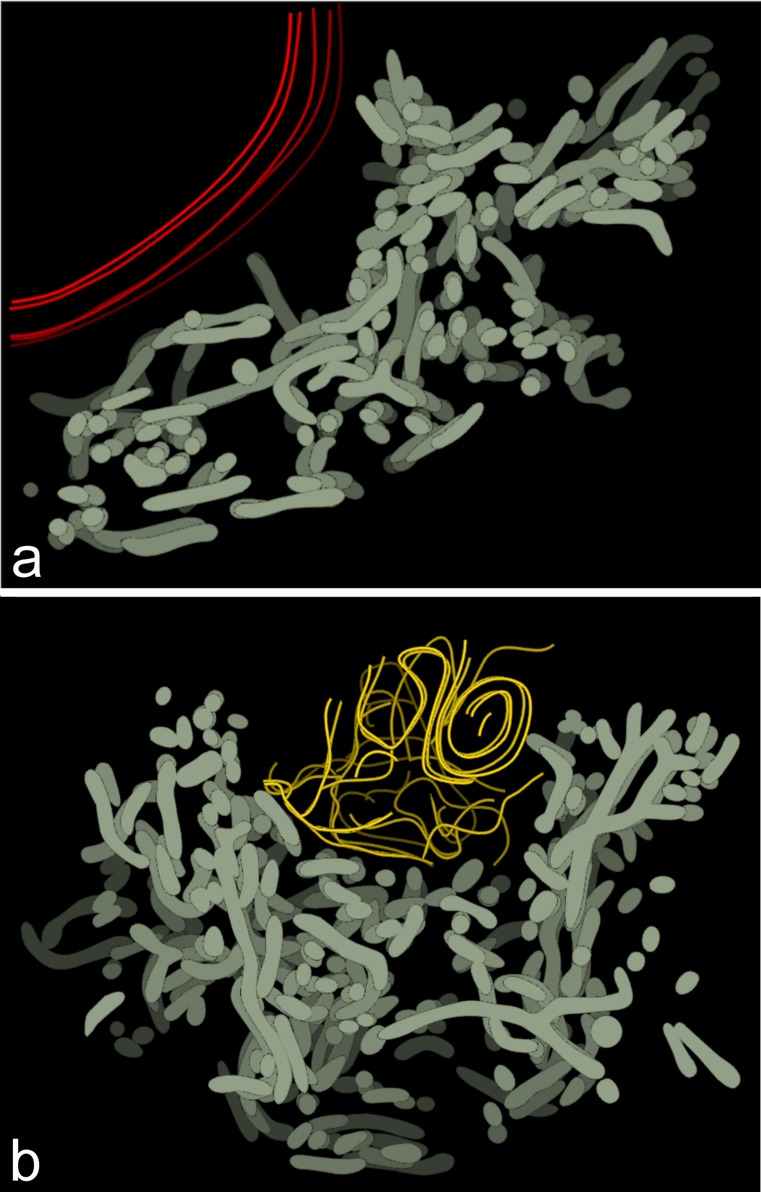
Computer-aided 3D reconstructions of dispersed Bbs. **a**,** b** Two representative 3D reconstructions of the mitochondrial networks in previtellogenic oocytes. Mitochondrial network (*gray*), elements of RER (*yellow*), nuclear envelope (*red*). For clarity of the images, colors of subsequent (deeper and deeper) levels (representing subsequent sections) have been depicted darker

The information that can be extracted from the examination of single, often incidental, sections of different (at different stages of morphogenesis) Bbs is not sufficient for the spatial reconstruction of this organelle assemblage. Therefore, to visualize exact relationships between Bbs constituents, we performed a computer-aided 3D reconstruction of serial ultrathin sections through the Bbs at two developmental stages: during their maximal expansion (at the bouquet stage of the meiotic prophase) and after the onset of their dispersion. Between 5 and 18 ultrathin sections were used in our reconstructions (Figs. 3, 4; animated 3D reconstructions are presented in supplementary materials; Movies 1–4).

Analysis of the obtained 3D images indicated that the central part of each fully developed Bb is occupied by an extensive mitochondrial network (Fig. 3, gray, and Movies 1, 2 in supplementary materials). Nuage accumulations (blue), Golgi complexes (magenta) and elements or RER (not shown in the reconstructions) are located on the periphery of this network (Figs. 2d, 3). This “core” organelle assemblage is surrounded by several small bean-shaped mitochondria (Fig. 3d–f). Careful analysis of EM micrographs used for 3D reconstructions indicated that some of these “isolated” mitochondria show signs of degradation (Fig. 3d–f, green); examples of mitochondria at various stages of degradation (mitochondria with swollen cristae and/or connected with dense lamellar bodies) are shown in Fig. 2d, f, g. Interestingly, morphologically altered mitochondria (mitochondrial fragments) were also observed in contact with the tips of “ramifications“ of the mitochondrial network (Fig. 3, green). Since 3D reconstructions from complete series of 18 sections are, in some places, not clear enough, we also present partial reconstructions of the most interesting parts of the analyzed Bbs (Fig. 3b, c, e, f).

Our reconstructions also showed that during early previtellogenesis (after the onset of Bb dispersion) mitochondria are still interconnected forming extensive networks (Fig. 4a, b; Movies 3, 4 in supplementary materials). It is interesting to note here that morphologically altered mitochondria, at this phase of oogenesis, are very rare.

### Do all germline cells differentiate into oocytes?

**Fig. 5:**
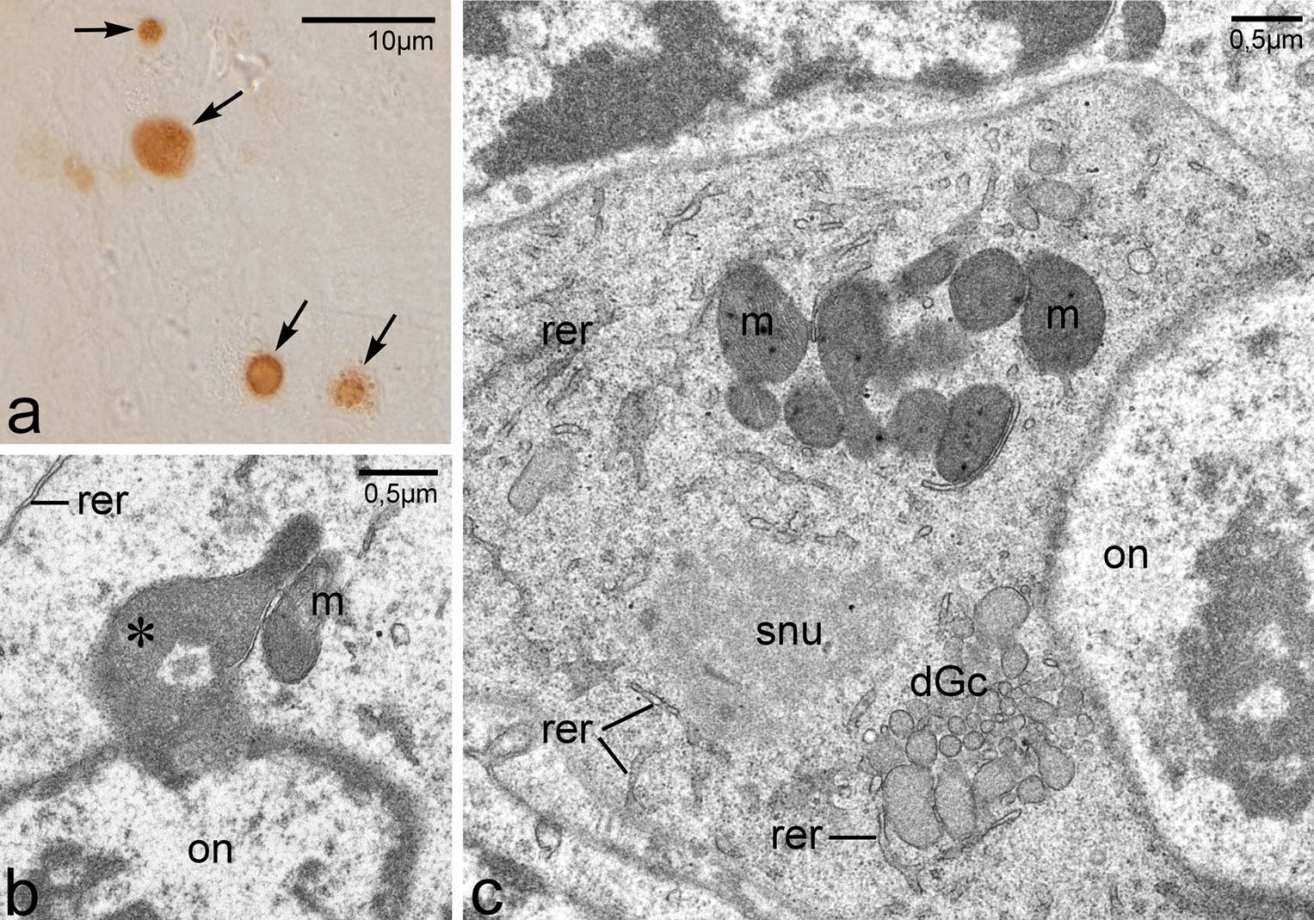
Apoptosis of germline cells. **a** Fragment of the germarium. TUNEL assay. Note apoptotic cells (*arrows*). **b, c** Fragments of apoptotic oocytes (TEM). Note altered morphology of Bb: dense mitochondria (*m*), transparent and swollen nuage accumulation (*snu*) and distended cisternae of Golgi complex (*dGc*). Elements of RER (*rer*), oocyte nucleus (*on*); blebs of the nuclear envelope filled with heterochromatin (**b**, *asterisk*)

It has been repeatedly suggested that animal oocytes overloaded with damaged mitochondria, i.e. severe mtDNA mutations, are eliminated via apoptosis during early stages of oogenesis (Buszczak and Cooley 2000; Peterson et al. 2003). In this context, we decided to test whether all germline cells present in the germaria of *Thermobia* survive and differentiate into oocytes. To address this issue, we performed whole-mount TUNEL assay. TUNEL staining allows labeling of the free ends of broken DNA molecules that are considered as indicators of cell death and apoptosis. We found that, in each analyzed germarium, there are at least 7–10 apoptotic germline cells (Fig. 5a). Ultrastructural (EM) analysis provided additional support for this finding, showing that several germline cells display characteristic features of apoptotic cells, e.g. condensed cytoplasm, blebs of the nuclear envelope filled with heterochromatin accumulations, distended Golgi cisternae (Fig. 5b, c). Finally, we counted apoptotic germline cells in serial sections also used for 3D reconstructions, and found that in these series 14 to 18 % of germline cells exhibit various signs of apoptosis. Interestingly, the Bbs of apoptotic oocytes also revealed altered morphology. They consist of large, dense mitochondria associated with transparent, “swollen” nuage accumulations (Fig. 5c). Thus, our results suggest that, in the germaria of *Thermobia*, a relatively large proportion of germline cells undergoes apoptosis.

## Discussion

### Morphogenesis of the Bb

Our detailed EM analyses indicated that in *Thermobia* the Bb starts to differentiate as early as in the Cbs. Theoretically, further morphogenesis of the Bb may include two processes: either fusion of mitochondria gathered around the nuage accumulation or proliferation of mitochondria (or a single mitochondrion) into a large and intricate network. We did not obtain decisive evidence for either of these possibilities. However, two observations, i.e. substantial increase of mitochondrial profiles (in ultrathin sections) in early meiotic oocytes as well as the presence of bifurcated mitochondria in the vicinity of the nuage, suggest that proliferation of mitochondria plays a role in the growth of the Bb.

Previously, we suggested that the localization of the Bb together with polar attachment of the bouquet chromosomes play a role in the early asymmetrization of *Thermobia* oocytes (Tworzydlo et al. 2014). In light of the present results, this hypothesis must be abandoned. The bouquet of chromosomes is formed distinctly later (during the meiotic prophase) than the Bb starts to arise, and, consequently, the initial asymmetry of the Cb is established. If so, it might be speculated that the Bb is responsible for the positioning of the chromosome bouquet, and that the mitochondrial network of the Bb delivers energy (ATP) necessary for the movement and/or attachment of chromosome telomeres. It is worth adding here that a similar association of the Bb and bouquet chromosomes has also been reported in early oocytes of *Xenopus* (Kloc et al. 2004b).

The polarity of the *Thermobia* oocytes is apparently transient and lasts only until the beginning of the previtellogenic growth. At this phase, the Bb disperses and gradually covers the entire perimeter of the germinal vesicle. Our 3D reconstructions clearly show that mitochondria of expanding (dispersing) Bb are still interconnected forming a large but loose network. During the expansion, mitochondria (or rather elements of the mitochondrial network) strongly elongate, suggesting that, at least in part, this process relies on the change of their shape. Finally, during advanced previtellogenesis, the mitochondrial network breaks down into individual mitochondria that uniformly populate the entire oocyte cytoplasm. We have not been able to recognize any particular region of the ooplasm in which mitochondria are enriched or to which mitochondria are preferentially transferred. This finding agrees with previous results showing that the oocytes and early embryos of *Thermobia* are devoid of any specific regions, for instance the germ or polar plasm (Rost 2004).

### Involvement of the Bb in the selective elimination of dysfunctional mitochondria

Our 3D reconstructions indicate that the central part of *Thermobia* Bb consists of a hyperfused mitochondrial network. These reconstructions additionally showed that the tips of ramifications of this network often remain in contact with morphologically altered mitochondria. Similarly altered mitochondria are also frequent in the cytoplasm surrounding the Bb. It is tempting to speculate that the altered mitochondria represent the morphological manifestation of the mitophagy. This idea agrees with the results of our experiment that showed significant differences in the membrane potential between the mitochondrial network on the one hand, and individual mitochondria on the other. In conclusion, we suggest that in *Thermobia* damaged mitochondrial units (likely to contain mutated mtDNAs), with lowered membrane potential “bud off” (“pinch off”) from the network and are subsequently eliminated in the cytoplasm. This assumption is consistent with the idea that mitochondria, containing deleterious components and exhibiting decreased membrane potential, are eliminated via autophagy, and that the deleterious components can be sorted inside mitochondria (reviewed by Westermann 2010; Youle and van der Bliek 2012; van der Bliek et al. 2013; Hoitzing et al. 2015). Obviously, the latter process is indispensable for inducing mitophagy of only (or almost only) dysfunctional mitochondria.

### The elimination of dysfunctional mitochondria in *Thermobia* oocytes is a two-step process

Several lines of evidence suggest that mitochondria with severely mutated mtDNA are selected and subsequently eliminated in the female germline (Fan et al. 2008; Stewart et al. 2008). Comparable processes have also been described in somatic cells (Palikaras et al. 2015) and during asymmetric divisions of stemlike cells (Katajisto et al. 2015). Although the mechanism of this purifying selection remains unknown, it has been proposed that it relies on the mtDNA bottleneck phenomenon (Hauswirth and Laipis 1982; Bergstrom and Pritchard 1998; Cao et al. 2007), and that mtDNA can be functionally tested during germ cell development (Cox and Spradling 2003; Stewart et al. 2008). Here, we suggest that this selection in the oocytes of *Thermobia* involves two steps. In the first one, the Bb is engaged (see below); during the second, the early meiotic oocytes are eliminated via apoptosis. This idea is strongly supported by results of our studies showing numerous apoptotic germline cells within the germaria. For the suggested two-step selection (filter), multiple divisions of germline stem cells, and a resulting surplus of early meiotic oocytes, are obviously required. Interestingly, this requirement is apparently fulfilled within the germaria of *Thermobia*, in which the germline stem cells are mitotically highly active and ultimately generate hundreds of oocytes (Tworzydlo et al., 2014). This has been shown earlier, consistent with our suggestion that only some of these oocytes complete the process of oogenesis and become fertilizable (Klag 1971).

## Conclusions

The results presented here clearly indicate that in *Thermobia* the Bb is not involved in the directional transport of organelles/macromolecules to certain oocyte region(s), and that polarity dictated by the position of this organelle assemblage is transient and lasts only until the beginning of a previtellogenic growth. Interestingly, similar transient polarity has also been described in the early oocytes of mouse (Kloc et al. 2008). Our results additionally suggest that in *Thermobia* the Bb might be implicated in the selective elimination of dysfunctional mitochondria. We propose the following scenario for this process:

- the number of mitochondria received by each Cb (generated by asymmetric divisions of the germline stem cells) is relatively low in accordance with the bottleneck hypothesis;
- mitochondria that come into contact with the nuage fuse and/or proliferate that leads to the formation of the Bb mitochondrial network; the others degenerate;
- dysfunctional mitochondrial units bud off from the network and are eliminated by autophagy.

The proposed scenario is well founded on the existing theoretical models of the mitochondrial quality control showing that fusion, fission and autophagy together increase mitochondrial functionality (reviewed by Hoitzing et al. 2015). However, further experimental studies are needed to support or reject this hypothesis.

Finally, we suggest that the participation of the Bb in the selective elimination of the dysfunctional mitochondria represents an ancestral function of this organelle assemblage. This is in line with previous observations indicating that the Bb is often present in oocytes that are devoid of the oosome/germ plasm (see Kloc et al. 2014 for a review), and, therefore, cannot be involved in the localization of proteins/mRNAs to a certain ooplasm region, as canonically accepted. It is tempting to speculate in this light that, during evolution of the Metazoa, the Bb was secondarily implicated in the transportation of the germ plasm constituents, but only in those invertebrate and vertebrate lineages in which the germ plasm evolved. This in turn agrees with the notion that postzygotic induction (epigenesis) of primordial germ cells represents an ancestral mechanism of germline specification, whereas the localization of maternal determinants (germ plasm) is a derived character that evolved independently (by convergence) in several animal lineages (see Extavour and Akam 2003; Ewen-Campen et al. 2010, 2013; Evans et al. 2014 for further discussion). Obviously, the above hypotheses should be further tested using non-model species representing basally branching and derived animal lineages.

## Electronic supplementary material

Below is the link to the electronic supplementary material.

Movie 1 3D reconstruction of the Bb in bouquet stage oocyte. Mitochondrial network (gray), nuage accumulations (*blue*), nuclear envelope (*red*) (AVI 1.74 mb)

Movie 2 3D reconstruction of the Bb in bouquet stage oocyte. Mitochondrial network (gray), degenerating mitochondria (*green*), nuage accumulations (*blue*), Golgi complex (*magenta*), nuclear envelope (*red*) (AVI 2.00 mb)

Movie 3 3D reconstruction of the dispersed Bb in previtellogenic oocyte. Mitochondrial network (*gray*), nuclear envelope (*red*) (AVI 1.53 mb)

Movie 4 3D reconstruction of the dispersed Bb in previtellogenic oocyte. Mitochondrial network (*gray*), elements of RER (*yellow*) (AVI 1.74 mb)
